# Biodegradation of Bisphenol A by *Sphingobium* sp. YC-JY1 and the Essential Role of Cytochrome P450 Monooxygenase

**DOI:** 10.3390/ijms21103588

**Published:** 2020-05-19

**Authors:** Yang Jia, Adel Eltoukhy, Junhuan Wang, Xianjun Li, Thet Su Hlaing, Mar Mar Aung, May Thet Nwe, Imane Lamraoui, Yanchun Yan

**Affiliations:** 1 Graduate School, Chinese Academy of Agricultural Sciences, Beijing 100081, China; jia_yang@outlook.com (Y.J.); adelaly@azhar.edu.eg (A.E.); wangjunhuan@caas.cn (J.W.); lixianjun1978@126.com (X.L.); thetsuhlaing11@gmail.com (T.S.H.); marmaraung1@gmail.com (M.M.A.); maythetnwe1@googlemail.com (M.T.N.); 2 Biotechnology Research Institute, Chinese Academy of Agricultural Sciences, Beijing 100081, China; imane.lamraoui1@gmail.com

**Keywords:** bisphenol A, *Sphingobium* sp, biodegradation, cytochrome P450, soil remediation

## Abstract

Bisphenol A (BPA) is a widespread pollutant threatening the ecosystem and human health. An effective BPA degrader YC-JY1 was isolated and identified as *Sphingobium* sp. The optimal temperature and pH for the degradation of BPA by strain YC-JY1 were 30 °C and 6.5, respectively. The biodegradation pathway was proposed based on the identification of the metabolites. The addition of cytochrome P450 (CYP) inhibitor 1-aminobenzotriazole significantly decreased the degradation of BPA by *Sphingobium* sp. YC-JY1. *Escherichia coli* BL21 (DE3) cells harboring pET28a-*bisdAB* achieved the ability to degrade BPA. The *bisdB* gene knockout strain YC-JY1Δ*bisdB* was unable to degrade BPA indicating that P450*_bisdB_* was an essential initiator of BPA metabolism in strain YC-JY1. For BPA polluted soil remediation, strain YC-JY1 considerably stimulated biodegradation of BPA associated with the soil microbial community. These results point out that strain YC-JY1 is a promising microbe for BPA removal and possesses great application potential.

## 1. Introduction

Bisphenol A (2, 2-bis (4-hydroxyphenyl) propane, BPA) is extensively used as a plasticizer or flame retardant in the production of synthetic polymers, such as polycarbonate plastics and epoxy resins [1,2,3,4]. The global BPA consumption closed to 7.7 million tons during 2015 and may reach 10.6 million tons by 2022. However, a quarter of the BPA was released into the environment during the process of production, transportation, or processing [5,6,7,8]. As a kind of synthetic endocrine-disrupting chemicals (EDCs), BPA would cause reproductive toxicity because of its estrogenic activity [9,10]. Previous studies revealed that BPA could also cause carcinogenicity [11], immunotoxicity [12], embryotoxicity [13], and transgenerational influence [14] to various species. Therefore, the elimination of BPA is essential to the ecosystem and public health.

Biodegradation of BPA by microorganisms has been proven to be a safe and economical approach to remove BPA from the environment [3,15]. Bacteria with the capacity to degrade several xenobiotics, including BPA, have been isolated and investigated. The first BPA degrading bacterium *Sphingomonas* sp. strain MV1 was isolated from the sludge of a plastic manufacturing facility [16]. BPA degrader *Sphingomonas bisphenolicum* strain AO1 was isolated, and the cytochrome P450 (CYP) monooxygenase gene involved in BPA degradation was investigated [17,18,19]. BPA degrader *Sphingobium* sp. BiD32 was isolated from activated sludge, and a *p*-hydroxybenzoate hydroxylase was hypothesized to be involved in BPA degradation [20]. Besides, several bacteria with biodegradation ability of BPA were isolated and explored, such as *Achromobacter xylosoxidans* strain B-16, *Cupriavidus basilensis* strain JF1, *C. basilensis* strain SBUG 290, and *Novosphingobium* sp. TYA-1 [21,22,23,24]. Although BPA degradation pathways have been extensively studied, the knowledge of metabolic mechanisms, including catalysts and genes, was still limited. Fungi and some nonspecific fungal enzymes were demonstrated to attack BPA, such as lignin peroxidase, laccases, manganese peroxidases, and versatile peroxidases [25,26,27]. CYP monooxygenases are a superfamily of enzymes that may be involved in the metabolism of xenobiotics. It was reported that *bisdA* and *bisdB* genes encoding ferredoxin and cytochrome P450 were responsible for BPA degradation in *Sphingomonas bisphenolicum* strain AO1 [17,19]. Extracellular laccases produced by some *Pseudomonas* showed BPA transformation capacity [28]. An ammonia monooxygenase in *Nitrosomonas europaea* was reported to be involved in BPA degradation [29]. Although some various biodegradation pathways information has emerged, the further efforts of genetic basis and mechanisms are imperative.

In the present study, a new BPA degrading bacterial strain *Sphingobium* sp. YC-JY1, which could utilize BPA as a sole carbon and energy source, was isolated and characterized. The biodegradation conditions were optimized, and the degradation pathway was proposed by detecting the degradation intermediates via high-performance liquid chromatography-quadrupole-time-of-flight tandem mass spectrometry (HPLC-QTOF-MS/MS). Furthermore, cytochrome P450 was heterogeneously expressed, and strain YC-JY1Δ*bisdB* was constructed to explore the role of the cytochrome P450 gene. In addition, the capacity of YC-JY1 to remediate BPA-contaminated soil was investigated.

## 2. Results and Discussion

### 2.1. Strain Isolation and Identification

**Figure 1:**
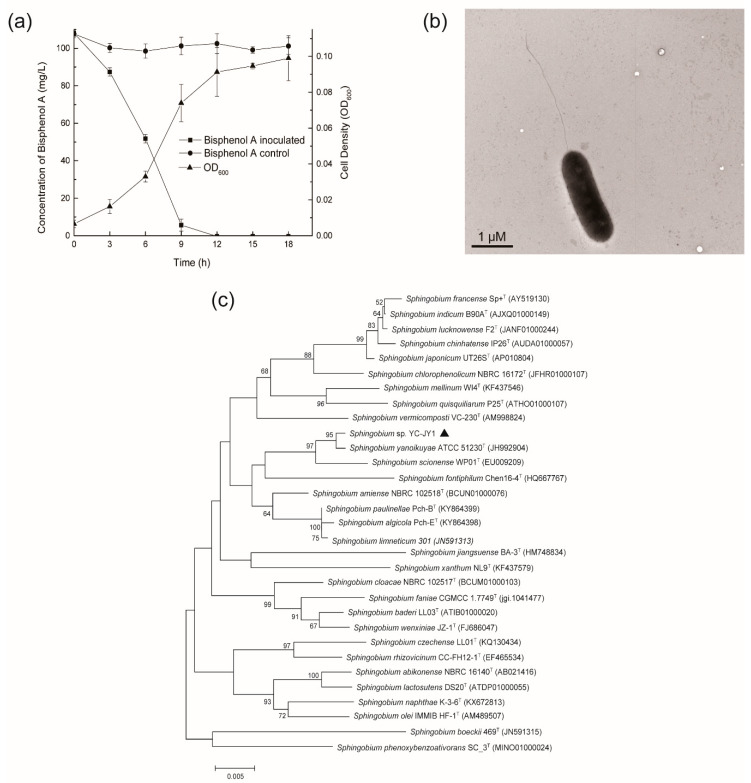
(**a**) Bisphenol A (BPA) degradation by strain YC-JY1 and growth curve. (**b**) The morphological characteristics of strain YC-JY1 observed by a transmission electron microscope. (**c**) Phylogenetic tree based on 16S rRNA gene sequence analysis of strain YC-JY1 and other *Sphingobium* genus strains. *Sphingobium* sp. YC-JY1 was marked using a dark triangle.

One strain capable of degrading BPA with high performance was isolated from the sludge samples by enrichment culture technique. Strain YC-JY1 degraded 100 mg/L BPA completely within 12 h under the condition of 30 °C and pH 7.0 (Figure 1a). The strain was rod-shaped with apical flagellum (Figure 1b). The16S rRNA gene sequences of strain YC-JY1 and other related type strains were used to construct the phylogenetic tree to identify strain YC-JY1. The neighbor-joining method was adopted for phylogenetic analysis. The phylogenetic tree (Figure 1c) showed that this strain was clustered with strain *Sphingobium yanoikuyae* ATCC 51230. The result of Biolog GEN Ⅲ microplate testing (Table S1) showed that it may be in the same species with *Sphingobium paucimobilis* B. Therefore, it suggested that strain YC-JY1 belongs to the genus *Sphingobium*, thus it was named as *Sphingobium* sp. YC-JY1. Several BPA-degrading bacteria have been isolated and investigated by researchers (Table 1). Most of the isolated BPA-degrading bacteria showed degradation activity at low concentration, and they took several days to degrade. *Sphingobium* sp. YC-JY1 completely degraded 100 mg/L BPA as sole carbon and energy source within 12 h. Obviously, *Sphingobium* sp. YC-JY1 was an excellent BPA-degrader with the ability to degrade a high concentration of BPA efficiently.

### 2.2. Effects of Environmental Factors on Biodegradation of BPA

**Figure 2:**
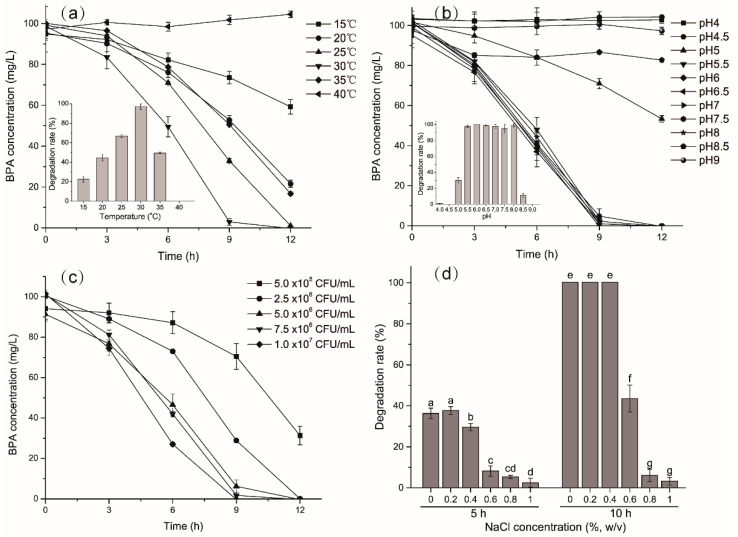
Effect of (**a**) temperature; (**b**) pH; (**c**) inoculums density and (**d**) NaCl concentration on biodegradation of BPA. The error bar value presents the standard deviation of triplicates. Inset shows BPA biodegradation efficiency at 9 h on different (**a**) temperature and (**b**) pH value. Different letters indicate statistically significant differences at *p* ≤ 0.05; similarly hereinafter.

The temperature, pH, inoculum density, and NaCl concentration are important factors influencing biodegradation significantly [9,33,34,35]. These factors not only impact on metabolism and growth of microorganisms, but also on the enzyme activity [36,37]. The effect of temperature on BPA biodegradation was shown in Figure 2a. The results indicated that the degradation efficiency increased with rising temperatures ranging from 15 to 30 °C, reached the maximum level at 30 °C and then the degradation efficiency gradually decreased with higher temperatures. The degradation efficiencies after 12 h incubation were 40.8% 78.4%, 99%, 100%, and 83.3% at the temperatures of 15, 20, 25, 30, and 35 °C, respectively. The degradation efficiencies of 100 mg/L BPA after 9 h of incubation were 22.4% (15 °C), 44% (20 °C), 66.9% (25 °C), 97.1% (30 °C), and 49.5% (35 °C). No degradation of BPA was observed at 40 °C, while the slightly increased concentration might be due to the evaporation of water. The result was in agreement with previous studies that higher temperatures within a certain range accelerated the degradation [9,21].

To detect the optimum pH value for BPA degradation by YC-JY1, 11 pH values from 4 to 9 were examined. The degradation efficiencies were 100% ranging from pH 5.5 to 8 after 12 h of incubation (Figure 2b). YC-JY1 showed decent degradation ability under both weak acidic and alkaline conditions, although BPA would be with low solubility in weak acidic conditions, and the bacterial growth and enzyme activity would be inhibited under weak alkaline condition.

Inoculums density would also affect the degradation of BPA. With the inoculum size from 5.0 × 10^5^ CFU/mL to 1.0 × 10^7^ CFU/mL, BPA was finished within 12 h while the degradation efficiency was 68.7% with the inoculum size of 5.0 × 10^5^ CFU/mL. The degradation efficiencies of 100 mg/L BPA after 9 h of incubation were 29.5%, 71.1%, 93.8%, 98.3%, and 100% with inoculum size 5.0 × 10^5^ CFU/mL, 2.5 × 10^6^ CFU/mL, 5.0 × 10^6^ CFU/mL, 7.5 × 10^6^ CFU/mL and 1.0 × 10^7^ CFU/mL, respectively (Figure 2c). These results indicated that higher cell density would increase the biodegradation efficiency, which was in accord with previous researches showing a synergistic effect of the biomass amount on biodegradation of BPA [9,21,38,39].

Salinity is another environmental factor that impacts biodegradation efficiency. In this study, NaCl concentrations ranging from 0% to 1% (*w/v*) were examined, and degradation efficiencies were determined after 5 h and 10 h incubation (Figure 2d). At 5 h, BPA biodegradation efficiency was not affected at 0.2% NaCl concentration but decreased at 0.4% NaCl concentration. At higher NaCl concentration s (0.6% to 1%), biodegradation was significantly inhibited. At 10 h, BPA was completely biodegraded at 0% to 0.4% NaCl concentration, and degradation efficiency decreased extremely at higher NaCl concentration. These results showed the degradation efficiencies were inhibited by adding NaCl, indicating that NaCl was a disadvantage of degradation.

### 2.3. Identification of Metabolites

To interpret the BPA biodegradation pathways and metabolic mechanisms in YC-JY1, HPLC-QTOF-MS/MS was employed to detect the metabolites. Intermediates of BPA biodegradation were identified with retention times of 3.873 min (2,3-bis(4-hydroxyphenyl)-1,2-propanediol, 3,4-BP, *m*/*z* 259.098 [M-H]^−^)(A), 5.709 min (4-hydroxybenzaldehyde, 4-HBD, *m*/*z* 121.03 [M-H]^−^) (B), 5.946 min (4′-hydroxyacetophenone, 4-HAP, *m*/*z* 135.046 [M-H]^−^) (C), 7.203 min (1,2-bis(4-hydroxyphenyl)-2-propanol, 1-BP, *m*/*z* 243.103 [M-H]^−^) (D), 12.236 min (BPA, *m*/*z* 227.108 [M-H]^−^) (E), 15.560 min (4,4′-dihydroxy-α-methylstilbene, 4-DM, *m*/*z* 225.092 [M-H]^−^) (F) (Figure S1). These proposed products were shown in Table 2. The proposed products 4-HAP and 4-HBD were verified using standards. 4-HAP and 4-HBD could be utilized by strain YC-JY1 as a sole carbon source, separately (data not shown). As time went on, a new peak with retention times of 6.89 min (*m*/*z* 211.077 [M-H]^−^) appeared in the sample after incubation for 9 h. After 24 h incubation, three peaks accumulated with retention time 3.873 min (*m*/*z* 259.098 [M-H]^−^), 6.89 min (*m*/*z* 211.077 [M-H]^−^), 8.086 min (*m*/*z* 213.056 [M-H]^−^). These substances were unknown.

**Figure 3:**
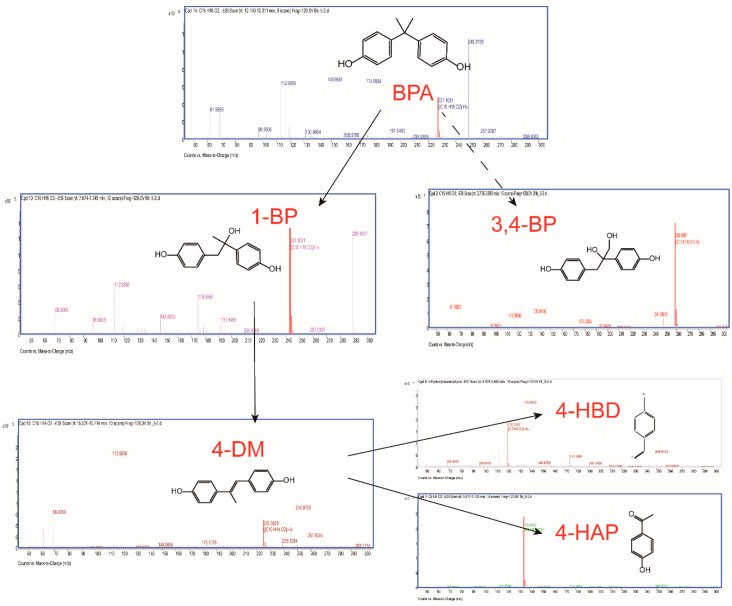
Proposed biodegradation pathway of BPA in *Sphingobium* sp. YC-JY1 based on HPLC-QTOF-MS/MS. BPA, bisphenol A; 1-BP, 1,2-Bis(4-hydroxyphenyl)-2-propanol; 3,4-BP, 2,3-Bis(4-hydroxyphenyl)-1,2-propanediol; 4-DM, 4,4′-Dihydroxy-α-methylstilbene; 4-HBD, 4-Hydroxybenzaldehyde; 4-HAP, 4′-Hydroxyacetophenone.

The strategy of the microbial transformation of BPA is diverse. *Mycobacterium* sp. transformed BPA by O-methylation into monomethyl and dimethyl ethers [40]. Sasaki et al. identified metabolites of BPA by GC-MS or LC-MS-MS, and the results indicated that 4-DM, 4-HAP, and 1-BP or 2-BP (2,2-bis(4-hydroxyphenyl)-1-propanol) were involved in the biodegradation pathway [18]. The metabolic pathway of BPA degradation in *Achromobacter xylosoxidans* strain B-16 was proposed according to the intermediates (*p*-hydroxybenzaldehyde, *p*-hydroxybenzoic acid, and *p*-hydroquinone) [21]. Based on nine identified intermediates, pathways of BPA degradation by spore laccase of *Bacillus* sp. GZB was proposed [41]. According to the identified metabolites and literature surveys, the metabolic degradation pathway of BPA by strain YC-JY1 was proposed, as shown in Figure 3. It was speculated that there were two routes in strain YC-JY1 to degrade BPA. In one pathway, BPA was converted into 1-BP, and then it was converted via 4-DM into 4-HBD and 4-HAP, which were then utilized by strain YC-JY1. In the other pathway, BPA was converted to 2-BP and then 3,4-BP. And 3,4-BP was accumulated in the medium. These results have a good consistency with some reports, and these intermediates also emerged in the processes of photocatalytic degradation and biotransformation of BPA [9].

### 2.4. Effect of Cytochrome P450 Inhibitor and BPA Degradation Activity of Cytochrome P450

**Figure 4:**
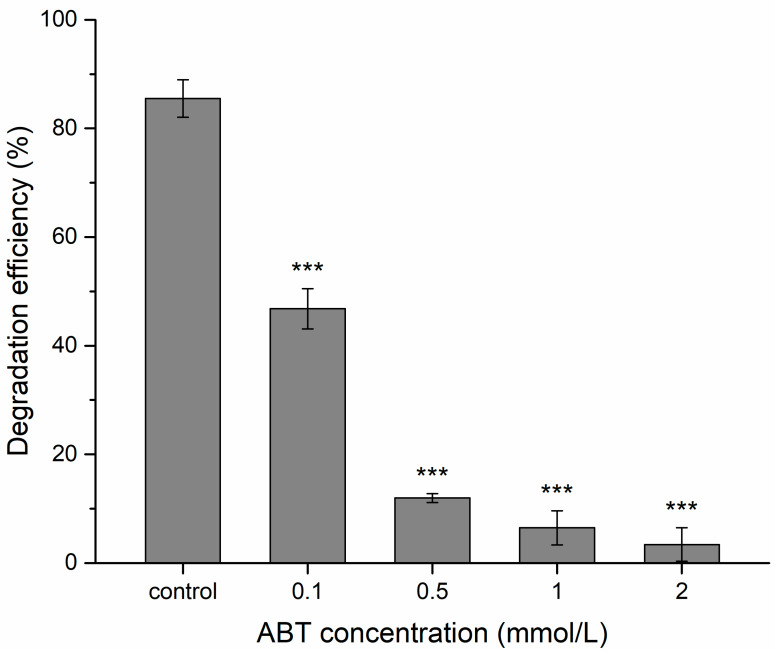
Effect of cytochrome P450 inhibitor ABT on BPA degradation by *Sphingobium* sp. YC-JY1 after 9 h of incubation. *** *p* < 0.001 indicates an extremely significant difference compared to the control.

1-aminobenzotriazole (ABT), a common inhibitor of CYP, was used to reduce the activity of CYP. It is often used to demonstrate whether a reaction is catalyzed by CYP enzymes [42,43,44]. In the presence of 0.1, 0.5, 1, and 2 mmol/L ABT, 46.8%, 12%, 6.5%, and 3.4% of BPA disappeared after 9 h of incubation, respectively, compared to 85.5% in cultures without ABT (Figure 4). ABT significantly inhibited BPA degradation, and the degradation efficiency decreased when ABT concentration rising, indicating that CYP was related to BPA degradation in strain YC-JY1. These findings indicated that the CYP is responsible for BPA degradation, which is in accord with some researches showing the involvement of CYP in xenobiotics transformation [37,44]. The presence of ABT demonstrated a slight decrease in BPA removal rate for *Chaetomium strumarium* G5I, *Thielavia arenaria* CH9, *Thielavia arenaria* HJ22 and *Thielavia arenaria* SM1(III), suggesting the effective contribution of CYP in the conversion [44].

**Figure 5:**
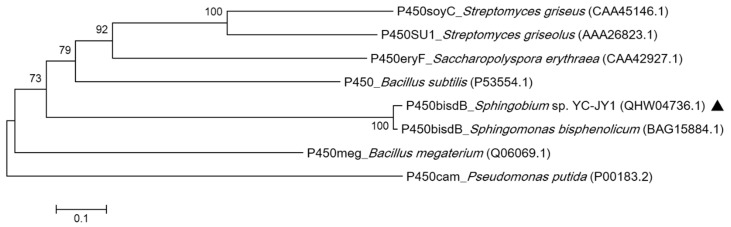
Phylogenetic analysis of P450*_bisdB_* from strain YC-JY1 and other bacterial cytochrome P450 proteins. The phylogenetic tree was constructed by the neighbor-joining method using MEGA 5.2 software. P450*_bisdB_* from *Sphingobium* sp. YC-JY1 was marked using a dark triangle. The accession numbers of the protein sequences were in brackets.

Sasaki et al. discovered that CYP, encoded by *bisd* in *Sphingomonas bisphenolicum* AO1 had the ability to degrade BPA. Based on genome sequence analysis, the *bisdA* and *bisdB* genes were cloned from YC-JY1. Phylogenetic analysis of P450*_bisdB_* encoded by *bisdB* gene of strain YC-JY1 and other bacterial cytochrome P450s is shown in Figure 5. P450*_bisdB_* was clustered with P450*_bisdB_* from *Sphingomonas bisphenolicum* AO1 and showed high similarity (97.9%). P450*_bisdB_* showed very low similarity with other P450s. The complete amino acid sequences of BisdA and BisdB proteins encoded by *bisdA* and *bisdB* genes of strain YC-JY1 were shown in the supplementary data, and the motifs of them were identified.

*E. coli* has been known to have some ferredoxins and ferredoxin reductases, flavodoxin, and flavodoxin reductases, which are needed by active P450s. It indicated that *E. coli* cells bearing *bisdB* might get the capacity of BPA degradation [19]. Consequently, the BPA-degradation capacity of *E. coli* BL21 (DE3) cells bearing pET28-*bisdB* or pET28-*bisdAB*, respectively, in LB medium supplied with BPA was investigated.

**Figure 6:**
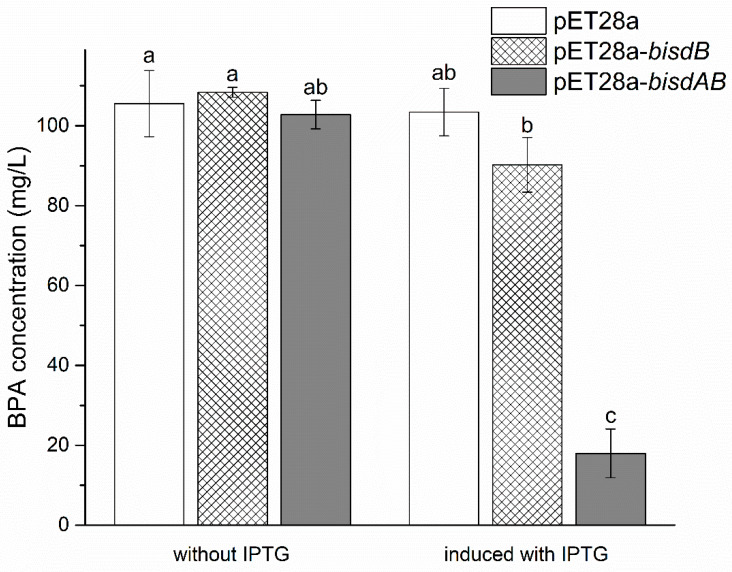
BPA degradation by pET28-*bisdB* and pET28-*bisdAB* recombinant cells after 24 h of incubation in Luria-Bertani (LB) medium. The different letters (a–c) represent significant differences at *p* ≤ 0.05.

BL21(DE3)-pET28a-*bisdB* cells converted less than 10 mg/L BPA after 24 h incubation with addition of IPTG, whereas BL21(DE3)-pET28a-*bisdAB* cells degraded about 85 mg/L BPA. While *E. coli* cells bearing *bisdB*-, *bisdAB*- without the addition of IPTG showed no BPA degradation capacity (Figure 6). It was opposite to the research results of Sasaki et al., stating BPA-degradation capacity decreased when IPTG was added into the medium [19]. Besides, ferredoxin of YC-JY1 was key for BPA degradation by P450*_bisdB_*, while the ferredoxin of *E. coli* could not meet the requirement well. The ferredoxin reductases of *E. coli* contributed to the degradation. However, activities of P450*_bisdB_* were not detected in the cell lysates (data not shown), indicating this recombinant P450*_bisdB_* may be in an unstable or inactive form, and the cells provided a relatively suitable environment for the reaction. A more appropriate expression system for P450*_bisdB_* was required. The metabolites of BPA transformed by *E. coli* cells bearing *bisdAB* were detected using HPLC-QTOF-MS/MS. 4-HBD, 4-HAP, 1-BP, 2-BP were identified in the metabolites (Figure S2). These results supported the proposed pathway via metabolites analysis.

### 2.5. Essential Role of CYP Gene for BPA Degradation in Strain YC-JY1

Since P450*_bisdB_* has shown to be able to remove BPA, it is curious to test whether YC-JY1 could metabolize BPA without the *bisdB* gene. A spontaneous mutant of strain AO1 without the *bisdAB* region lost the BPA degradation ability [19]. However, that cannot be ruled out by the influence of other lost genes. Thus, a knockout strain was constructed to test the role of *bisdB* in the BPA metabolism of strain YC-JY1. Strain YC-JY1Δ*bisdB,* which grew as well as YC-JY1 in the LB medium, lost its ability of BPA degradation in the TEM medium (Figure S3) with no visible growth, implying that the *bisdB* gene is essential for the catabolism of BPA and it initiated the degradation of BPA in strain. Knockout of *bisdB* intuitively and rigorously demonstrated the importance of *bisdB* in the degradation process of BPA.

### 2.6. Effect of Inoculated YC-JY1 on Remediation of BPA-Contaminated Soil

**Figure 7:**
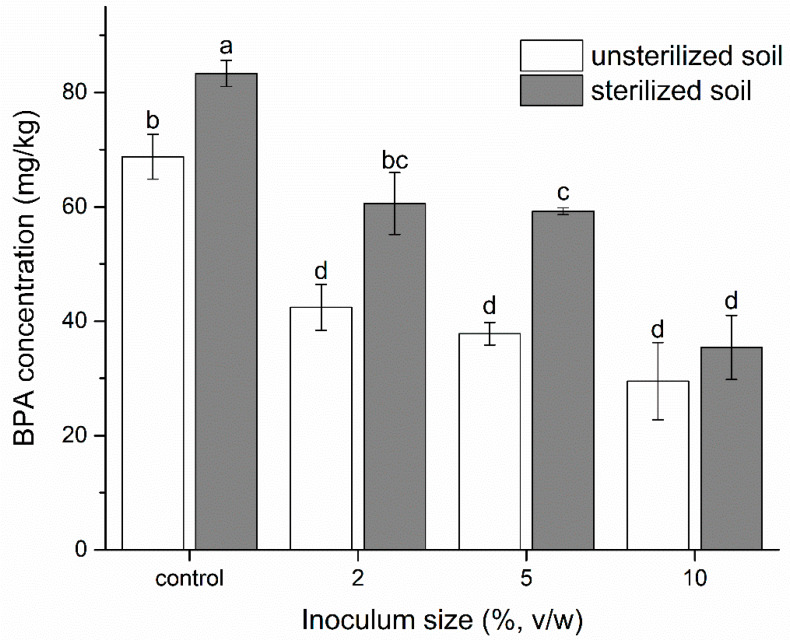
Decontamination of BPA-contaminated soil by inoculating *Sphingobium* sp. YC-JY1. The different letters (a–d) represent significant differences at *p* ≤ 0.05.

The microbial inoculation of strain YC-JY1 in BPA-contaminated soils was conducted to explore the influence of strain YC-JY1 on moderating BPA contamination. Results showed that strain YC-JY1 possessed strong soil remediation capabilities. As shown in Figure 7, the residual amount of BPA was 29.5 mg/kg (unsterilized group) and 35.4 mg/kg (sterilized group) after inoculating 5.0 × 10^7^ CFU/g YC-JY1 and incubating for 2 days. In both groups, BPA residues decreased with the increase of the inoculum amount and BPA residues in the unsterilized group were significantly less than that in the corresponding sterilized group, implying that, there may be some indigenous BPA degraders or an associated metabolism between YC-JY1 and resident microbiota. The soil environment is so complex that the degradation efficiency would be affected by many aspects, e.g., soil constitution, humidity content, microbial species, etc. Cooperative metabolism in microbial communities or consortia would promote biodegradation efficiency compared with the biodegrading bacterium alone [45]. Microbial interaction metabolism based on the cross-feeding with intermediates of the BPA degradation was a considerable factor for soil remediation. The cooperation enabled the microbial community to efficiently utilize carbon and energy from BPA [45]. It has been reported that supplementation with *Sphingomonas bisphenolicum* AO1 was able to significantly improve the BPA decomposition activity of the microbial community in soil [46]. *Pseudomonas* sp. BP-14, *Pseudomonas* sp. BP-15, and strain No. 24A without BPA degradation ability could accelerate the degradation of BPA by *Sphingomonas* sp. BP-7 [30]. During the initial phase of BPA degradation, inoculated strain YC-JY1 with CYP would play a dominant role, while other microbes, which lacked genes responsible for initial BPA degradation but possessed the lower pathway of BPA degradation, would promote the transformation process. This cooperative substrate cross-feeding between BPA-degrading microbes and non-degrading microbes accelerated the pollutant removal in the environment. Based on its potential application, strain YC-JY1 is expected to be a candidate as a biodegradable cleanser for environmental remediation.

## 3. Materials and Methods

### 3.1. Chemicals and Medium

Bisphenol A (BPA) (>99% purity) was purchased from Shanghai Macklin Biochemical Co., Ltd. 1-aminobenzotriazole (ABT) (98% purity) was from J&K Scientific. HPLC grade acetonitrile was from Thermo Fisher Scientific Inc. Trace element medium (TEM) and Luria-Bertani (LB) medium were used for enrichment and purification of isolated bacteria and bacteria culture. The detailed information of media was shown in the supplementary data. The pH was adjusted to approximately 7, and agar (1.6%) was added into LB and TEM media to prepare solid media. The media were sterilized at 121 °C for 20 min. Antibiotics were added to the medium as necessary.

### 3.2. Strain Isolation and Identification

The sludge sample was collected from a river in Guangdong Province, China (23°17′53.38″ N 116°20′21.41″ E). The TEM medium containing BPA (range from 50 mg/L to 300 mg/L) was used for enrichment and purification of BPA degradation strain. Approximately 5 g of soil was added into 50 mL TEM medium in a 250 mL-Erlenmeyer flask, supplied with 50 mg/L of BPA. The culture was incubated at 180 rpm and 30 °C for 5 days. 5 mL of the culture was transferred into 50 mL fresh TEM medium with 100 mg/L BPA. These steps were further repeated 4 times until the BPA concentration reached 300 mg/L (BPA concentration increased 50 mg/L each time).

Strain YC-JY1, which showed an excellent capability to degrade BPA was isolated. The cells of YC-JY1 were observed using a transmission electron microscope. It was identified using the Biolog GEN Ⅲ microplate protocol. The detailed information of the protocol was shown in the supplementary data. The partial 16S rRNA gene was amplified by polymerase chain reaction (PCR) with the universal primers 27F and 1492R (Table S2). BLAST was employed for homology search, and 16S rRNA gene sequences of standard bacterial strains were downloaded from a List of Prokaryotic names with Standing in Nomenclature (LPSN, http://www.bacterio.net/). The phylogenetic tree was built to analyze the evolution using MEGA (version 5.2, Tempe, AZ, USA).

### 3.3. Preparation of the Bacterial Suspension

Strain YC-JY1 was pre-cultured in LB medium at 30 °C for 12 h, harvested by centrifugation at 5000 rpm for 5 min and then washed twice with TEM medium. The bacteria were then resuspended in TEM medium to set optical density at 600 nm (OD_600_) of 0.8. Colony-forming units (CFU/mL) of this suspension were quantified by the dilution plate count technique. For batch experiments, 100 μL of the suspension was inoculated into 10 ml of TEM with BPA to obtain a final cell density of approximately 5.0 × 10^6^ CFU/mL for BPA biodegradation following studies.

### 3.4. Effects of Environmental Factors on BPA Biodegradation

To investigate the impacts of environmental factors on the degradation of BPA, a series of batch experiments about temperature, pH, inoculum density, and NaCl concentration were conducted. The incubation temperatures were set as 15–40 °C at pH 7. The initial pH of the TEM medium was adjusted to 4–9 at 30 °C. Inoculum size (5.0 × 10^5^ CFU/mL to 1.0 × 10^7^ CFU/mL) and NaCl concentration (0%–1%, *w/v*) were carried out at 30 °C, pH 7. The incubation was performed in 50 mL-Erlenmeyer flasks at 180 rpm in the dark. BPA residues in culture media were detected by high-performance liquid chromatography (HPLC) (Agilent 1200, Palo Alto, CA, USA).

### 3.5. Identification of Metabolites

To investigate the BPA degradation pathway in strain YC-JY1, the BPA metabolites after incubating for 0 h, 3 h, 6 h, 9 h, 12 h, and 24 h were analyzed. The cell cultures (10 mL) were extracted twice using an equal volume of ethyl acetate and then concentrated by rotary evaporator (R-215, Buchi, Switzerland). The residues were dissolved in 500 μL HPLC-grade acetonitrile and filtered using 0.22 μm membrane (Millipore, Bedford, MA, USA) for analysis. HPLC-QTOF-MS/MS (Agilent 6500, Agilent 6545, Palo Alto, CA, USA) was employed to detect the samples.

### 3.6. Cytochrome P450 Inhibitor Experiment

ABT was used as a CYP inhibitor to investigate the role of CYP in BPA degradation in this study. ABT was added into TEM medium, and final ABT concentrations were 0.1, 0.5, 1, and 2 mmol/L, respectively. Then, strain YC-JY1 was pre-incubated with ABT for 2 h, followed by the addition of BPA (100 mg/L). These cultures were further incubated for 9 h, and BPA residues were measured by HPLC. The cultures without ABT were served as control. Each treatment was conducted in triplicate.

### 3.7. Cloning and Expression of bisdB and bisdAB

The genomic DNA of strain YC-JY1 was extracted using a bacterial genomic DNA extraction kit (TaKaRa, Dalian, China). Based on genome sequence analysis of strain YC-JY1, potential BPA degradation genes *bisdA* and *bisdB* were proposed. Gene *bisdB* was amplified with primers bisdB-F and bisdB-R. The *bisdAB* fragment was amplified from strain YC-JY1 using primers bisdAB-F, bisdAB-R (Table S2). The PCR products were purified using a DNA fragment purification kit (TaKaRa) and ligated into the pET28a(+) vectors after digestion to obtain the plasmids pET28a-*bisdB* and pET28a-*bisdAB*. The plasmids were then transformed into *E. coli* BL21(DE3). BPA transformation products by *E. coli* (pET28a-*bisdAB*) were analyzed using HPLC-QTOF-MS/MS.

### 3.8. Gene Knockout of bisdB

Plasmid pEX18Tc-*bisdB* for gene knockout was constructed by fusing PCR products of the kanamycin resistance gene (*kan*), an upstream fragment (uf) of bisdB gene amplified with primers bisdBup-F and bisdBup-R, and a downstream fragment (df) amplified with primers bisdBdown-F and bisdBdown-R to the *Eco*R I and *Hin*d Ⅲ digested pEX18Tc using the Uni Seamless Cloning and Assembly Kit (Transgen, Beijing, China). The plasmid with uf-*kan*-df fragment was then transformed into *E. coli* SM10 λpir before its conjugation with strain YC-JY1. The double-crossover recombinants of strain YC-JY1Δ*bisdB* were screened on LB plates containing nitrofurantoin, kanamycin, and 15% (*w*/*v*) sucrose.

### 3.9. Remediation of BPA-Contaminated Soil

The effects of inoculated strain YC-JY1 on BPA residues in soil were evaluated. The soil collected from the garden was air-dried and sieved (0.45 mm). The soil experiment was set in 2 groups (unsterilized and sterilized soil). All soils were spiked with 100 mg/kg BPA and inoculated with a different final cell density of strain YC-JY1 as following, 0 CFU/g, 1.0 × 10^7^ CFU/g, 2.5 × 10^7^ CFU/g and 5.0 × 10^7^ CFU/g. These treatments were conducted in triplicate and incubated at 30 °C for 2 days in the dark. BPA residues were extracted from the soil using 10 mL acetonitrile shaking in the dark for 2 h. Then the samples were placed in 4 °C for 2 h. The BPA residues were detected by HPLC.

### 3.10. Analytical Methods and Statistical Analysis

Cell growth was measured as OD_600_ by a UV-Visible spectrophotometer (Thermo Scientific, Wilmington, DE, USA). The concentration of BPA was detected by HPLC (Agilent 1200, USA) equipped with an Eclipse XDB-C18 column (4.6 × 150 mm, 5 μm) and a visible ultraviolet detector. The mobile phase was acetonotrile and 0.1% formic acid in water (70:30). The flow rate was 1 mL/min with an injection volume of 2 μL and detecting wavelength of 220 nm.

An Agilent 6500 HPLC system and Agilent 6545 QTOF mass spectrometer equipped with an Eclipse XDB-C18 column (4.6 × 150 mm, 5 μm) were used to detect the metabolites of BPA. The mobile phase was acetonitrile (A) and 0.1% formic acid in water (B). A gradient run was used as follows: 0–5 min, 30% A; 5–6 min, 30–50% A; 6–11 min, 50% A; 11–12 min, 50–70% A; 12–13 min 70–90% A; 13–18 min, 90% A; 18–20 min, 90–50% A; 20–22 min, 50–30% A; 22–25 min, 30% A. The flow rate was 0.5 mL/min with an injection volume of 2 μL. The column oven temperature was maintained at 30 °C. The ESI ion source was operated in negative mode. Operation conditions were set as follows: The gas temperature was 325 °C; gas flow was 6 L/min; nebulizer pressure was 35 psig; sheath gas temperature was 350 °C; sheath gas flow was 11 L/min; scan model and negative ions in the mass range of 50 to 500 *m*/*z* was adopted; capillary voltage was 3.5 kV. Mass Hunter (version B.07.00, Agilent, Palo Alto, CA, USA) was employed to analyze the data.

The data analyses were performed by *t*-test using SPSS software (version 20, Chicago, IL, USA). The statistical significance was accepted at *p* < 0.05.

### 3.11. Accession Numbers

The 16S rRNA gene sequence of *Sphingobium* sp. YC-JY1 was deposited in Genbank under the accession number MN165536. The *bisdA* and *bisdB* gene sequences were deposited in Genbank with access number MN171527. Strain YC-JY1 was deposited in the China General Microbiological Culture Collection Center (Beijing, China) under the accession CGMCC No. 16352.

## 4. Conclusions

An efficient BPA-biodegrading bacterium *Sphingobium* sp. YC-JY1 was isolated and identified. The BPA degradation efficiency was affected by a few factors (e.g., temperature, pH, inoculum size, and NaCl concentration). The biodegradation pathway was proposed based on the analysis of the metabolites. It was certified that P450*_bisdB_* was the essential initiator for BPA metabolism in strain YC-JY1. Strain YC-JY1 considerably stimulated biodegradation of BPA in BPA-contaminated soil, showing YC-JY1 is a potential candidate for remediation of the BPA-contaminated environment.

## Figures and Tables

**Table 1:** Biodegradation of BPA by various microorganisms.

Microorganism	Resources	Degradation Efficiency	References
*Sphingomonas* sp. strain MV1	sludge	100%, 10 g/L, 4 days	[16]
*Sphingomonas bisphenolicum* strain AO1	soil	100%, 115 mg/L, 117 h (without other carbon sources); 100%, 115 mg/L, 6 h (with glucose)	[18]
*Achromobacter xylosoxidans* B-16	wastes compost leachate	100%, 3 mg/L, 4.5 days	[21]
*Sphingomonas* sp. strain BP-7	offshore seawater	>95%, 114 mg/L, 40 days	[30]
*Cupriavidus basilensis* JF1	fixed bed reactor	50%, 34.2 mg/L, 144 days	[23]
*Bacillus* sp. GZB	sediment	92.9%, 5 mg/L, 96 h	[9]
*Sphingobium sp.* BiD32	sludge	100%, 1 mg/L, 4.2 h	[31]
*Nitrosomonas europaea* ATCC 19718	NR ^1^	>50%, 1 mg/L, 5 days	[32]

^1^ NR means not reported.

**Table 2:** Degradation metabolites of BPA by strain YC-JY1 using HPLC-QTOF-MS/MS.

Proposed Products	RT (min)	M (*m*/*z*)	MS/MS (*m*/*z*)	Molecular Formula	Structure
1,2-Bis(4-hydroxyphenyl)-2-propanol	7.171~7.212	243	135, 210, 225	C_15_H_16_O_3_	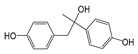
4,4′-Dihydroxy-α-methylstilbene	15.557~15.565	225	69	C_15_H_14_O_2_	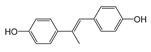
4-Hydroxybenzaldehyde	5.700~5.709	121	92	C_7_H_6_O_2_	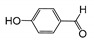
4′-Hydroxyacetophenone	5.931~5.947	135	92, 120	C_8_H_8_O_2_	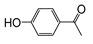
2,3-Bis(4-hydroxyphenyl)-1,2-propanediol	3.839~3.873	259	62, 135	C_15_H_16_O_4_	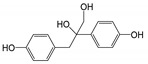

## Supplementary Materials

Supplementary materials can be found at https://www.mdpi.com/1422-0067/21/10/3588/s1, Table S1: Layout of assays for MicroPlate (GEN III). Table S2: Strains, plasmids, and primers used in this study. Figure S1: HPLC-TOF-MS/MS chromatograms of intermediates during the biodegradation of BPA from 0 h to 24 h. Figure S2: The chromatogram and mass spectra of primary ions for BPA degradation by E. coli (pET28a-*bisdAB*) intermediates analyzed by HPLC-TOF-MS/MS. Figure S3: BPA degradation by strain YC-JY1 and strain YC-JY1Δ*bisdB* in TEM medium.
