# Supplementary material for: Biodegradation of Bisphenol A by Sphingobium sp. YC-JY1 and the Essential Role of Cytochrome P450 Monooxygenase
Source: Int J Mol Sci. 2020 May 19;21(10):3588. doi: 10.3390/ijms21103588 (PMC7278973; doi:10.3390/ijms21103588)
Supplement: Supplementary file 1 [file ijms-21-03588-s001.pdf]

Supplementary data

Biodegradation of bisphenol A by *Sphingobium* sp. YC-JY1 and the essential role of cytochrome P450 monooxygenase

Yang Jia[1], Adel Eltoukhy[1], Junhuan Wang[1], Xianjun Li[1], Thet Su Hlaing[1], Mar Mar Aung[1], May Thet Nwe[1], Imane Lamraoui[2], Yanchun Yan[1,*]

[1] Graduate School, Chinese Academy of Agricultural Sciences, Beijing 100081, China

[2] Biotechnology Research Institute, Chinese Academy of Agricultural Sciences, Beijing 100081, China

[*]Correspondence: Yanchun Yan

Email: yanyanchun@caas.cn

Telephone: 010-82109685

Media

Luria-Bertani (LB) medium consists of 10.0 g peptone, 5.0 g yeast extract, and 10.0 g NaCl in 1.0-liter deionized water. Trace element medium (TEM) was used for enrichment and purification of isolated bacteria, and it contained (per liter) $(NH_4)_2SO_4$, 2.0 g; $Na_2HPO_4\cdot12H_2O$, 1.5 g; KH_2PO_4, 1.5 g; $MgSO_4\cdot7H_2O$, 0.2 g; $CaCl_2\cdot2H_2O$, 0.01 g, and trace elements (mg/L): $FeSO_4\cdot7H_2O$ (5), $ZnSO_4\cdot7H_2O$ (0.22), $CuSO_4\cdot5H_2O$ (0.03), $Na_2MoO_4\cdot2H_2O$ (0.02), $MnSO_4\cdot2H_2O$ (1.43), $CoSO_4\cdot7H_2O$ (0.12), $Na_2WO_4\cdot2H_2O$ (0.023).

Microbial identification using Biolog

Isolated bacterium was identified using the Biolog GEN III MicroPlate protocol. The GEN III MicroPlate™ test panel provided a standardized micromethod using 94 biochemical tests. The cells were freshly regrown on LB plate in order to avoid the loss of viability and metabolic vigor which is typical of most organisms at stationary phase. Using inoculation fluid (IF), inoculums of each target cell were prepared with Protocols A (IF-A catalog no. 72401) and B (IF-B catalog no. 72402) at turbidity range of 95–98 % T.

An 8-channel automated pipettor was used to dispense 100 μL of the suspension into each of the wells in the MicroPlate (Catalog no. 1030). The wells (Table S2) contain 71 carbon source utilization assays (columns 1–9) and 23 chemical sensitivity assays (columns 10–12); hence, they can be identified at the species levels based on the ‘‘Phenotypic Fingerprint’’ of the microorganisms provided by the test panel. These MicroPlates were placed in Omnilog reader, where they were read using Biolog’s Microbial Identification Systems software. Identified microbes were recorded.

Protein sequences

>BisA sequence

MPHIQVTTRDGEIRELDVAASGFLMEALRDANIDGVEAICGGCCSCATCHVYIDAAPAGT

LPPVSSDEEMLLSGLVSTPGRSRLSCQIPVTAELDGLKLTIPPDS

Conserved cysteine residues are indicated by bold boxes.

>BisB sequence

MNPQTLPVFPDLDIFSPEYACNREKYAARALRDYPLHFYKPLNMWIVSKHKDVRSALFTP

QVFSSVAFGLLPPPDDIAPRVPDLYTDVHLPSMDPPEHTKLRVPVQQALLPGRLVGKDEVV

RRIANELIDTFIDKGECDLLHDFSYKLALYLIVDMLGLPKERAEDYHRWSNCFFQLFTPKV

PERADARFFVPMPEEVLRQIWEDLAEANDYLREVVENLDRNPGNNMLSNLLQLREPDGS

RTITISANVRNALEFGAAGHDTTATLIAHLTYFVLTTPDLKDTLTEDPSLIPAAISETLRRRGS

VDGLFRRTLSDVELCGQKIESGSIVYLDLTAANLDPDVFPEPETFRLNRDNIKEMVSFGYG

RHVCAGQYLSRIEAKAAYEELMRRIPNMRLADGFKLEYMPSVATTVLKGLPLVWDKN

The dashed line indicates the oxygen-binding region with a conserved threonine residue (bold box). The solid line indicates the heme-binding region, including the heme-binding cysteine residue (bold box).

Table S1 Layout of assays for MicroPlate (GEN III)

A1<br>Negative control	A2<br>Dextrin	A3<br>D-Maltose	A4<br>D-Trehalose	A5<br>D-Celloblose	A6<br>Gentiblose	A7<br>Sucrose	A8<br>D-Turanose	A9<br>Staychose	A10<br>Positive control	A11<br>pH 6	A12<br>pH 5
B1<br>D-Rafflinose	B2<br>α-D-Lactose	B3<br>D-Melibiose	B4<br>β-Methyl-D-Glucoside	B5<br>D-Salicin	B6<br>N-Acetyl-D-Glucosamine	B7<br>N-Acetyl-β-Mannosamine	B8<br>N-Acetyl-D-Galactosamine	B9<br>N-Acetyl Neuraminic acid	B10<br>1 % NaCl	B11<br>4 % NaCl	B12<br>6 % NaCl
C1<br>α-D-Glucose	C2<br>D-Mannose	C3<br>D-Fructose	C4<br>D-Galactose	C5<br>3-Methyl glucose	C6<br>D-Fucose	C7<br>L-Fucose	C8<br>L-Rhamnose	C9<br>Inosine	C10<br>1 % Sodium lactate	C11<br>Fusidic acid	C12<br>D-Serine
D1<br>D-Sorbitol	D2<br>D-Mannitol	D3<br>D-Arabitol	D4<br>Myo-inositol	D5<br>Glycerol	D6<br>D-Glucose-6- PO4	D7<br>D-Fructose-6-PO4	D8<br>D-Aspartic acid	D9<br>D-Serine	D10<br>Troleandomycin	D11<br>Rifamycin SV	D12<br>Minocycline
E1<br>Gelatin	E2<br>Glycl-L-Proline	E3<br>L-Alanine	E4<br>L-Arginine	E5<br>L-Aspartic acid	E6<br>L-Glutamic acid	E7<br>L-Histidine	E8<br>L-Pyroglutamic acid	E9<br>L-Serine	E10<br>Lincomycin	E11<br>Guanidine HCl	E12<br>Niaproof 4
F1<br>Pectin	F2<br>D-Galacturonic acid	F3<br>L-Galactonic acid lactone	F4<br>D-Gluconic acid	F5<br>D-Glucuronic acid	F6<br>Glucuronamide	F7<br>Mucic acid	F8<br>Quinic acid	F9<br>D-Saccharic acid	F10<br>Vancomycin	F11<br>Tetrazolium Violet	F12<br>Tetrazolium Blue
G1<br>p-Hydroxy-Phenlyacetic Acid	G2<br>Methyl Pyruvate	G3<br>D-Lactic acid methyl ester	G4<br>L-Lactic acid	G5<br>Citric acid	G6<br>α-Keto-Glutaric acid	G7<br>D-Malic acid	G8<br>L-Malic acid	G9<br>Bromo-Succinic acid	G10<br>Nalidixic acid	G11<br>Lithium Chloride	G12<br>Potassium tellurite
H1<br>Tween 40	H2<br>γ-Amino-Butyric acid	H3<br>α-Hydroxy-Butyric acid	H4<br>β-Hydroxy-D,L Butyric acid	H5<br>α-Keto-Butyric acid	H6<br>Acetoacetic acid	H7<br>Propionic acid	H8<br>Acetic acid	H9<br>Formic acid	H10<br>Aztreonam	H11<br>Sodium butyrate	H12<br>Sodium bromate

Table S2 Strains, plasmids and primers used in this study

Strains, plasmids and primers	Description	Source
***Sphingobium* sp. strains**		
YC-JY1	wild-type bisphenol A degrader; Nit$^{r\ a}$	this study
YC-JY1Δ*bisdB*	YC-JY1 mutant with *bisdB* gene replaced with kanamycin resistance gene	this study
***E. coli* strains**		
Trans1-T1	F^{-}φ80(*lacZ*)ΔM15Δ*lac*X74*hsd*R(r_k^-, m_k^+)Δ*rec*A1398*end*A1*ton*A	TransGen
BL21(DE3)	host strain for expression vectors; F^- *omp*T *hsd*S_B(r_B^- m_B^-) *gal dcm*(DE3)	Tiangen
SM10λpir	donor strain for conjugation, thi thr leu tonA lacY supE recA∷RP4-2-Tc∷Mu	Zomanbio
**plasmids**		
pET28a(+)	expression vector; Kan$^{r\ b}$	Novagen
pEX18Tc	gene knockout vector, *oriT*, *sacB*, Tc$^{r\ c}$	Miaolingbio
pET28a-*bisdB*	pET-28a(+) derivative carrying *bisdB*	this study
pET28a-*bisdAB*	pET-28a(+) derivative carrying *bisdA* and *bisdB*	this study
pEX18Tc-*bisdB*	pEX18Tc derivative carrying *bisdB*	this study
**Primers**		
27F	5′ - AGAGTTTGATCCTGGCTCAG-3′	this study
1492R	5′- GGTTACCTTGTTACGACTT-3′	this study
bisdB-F	5′-GCGC<u>GAGCTC</u>ATGAACCCTCAGACACTGC-3′[d]	this study
bisdB-R	5′-GCGC<u>AAGCTT</u>GTTTTTGTCCCAGACCAGC-3′	this study
bisdAB-F	5′-GCGC<u>GAGCTC</u>ATGCCTCATATCCAAGTGACT-3′	this study
bisdAB-R	5′-GCGC<u>AAGCTT</u>GTTTTTGTCCCAGACCAGC-3′	this study
bisdBup-F	5′-GCTATGACCATGATTACGAAGATACTGATCAAGCCGGTGCG-3′	this study
bisdBup-R	5′-CCCGTTGAATATGGCTCATGTTCGGATTCCCGCTC-3′	this study
kan-F	5′-ATGAGCCATATTCAACGGGA-3′	this study
kan-R	5′-TTAGAAAAACTCATCGAGCATCA-3′	this study
bisdBdown-F	5′-TGATGCTCGATGAGTTTTTCTAAGCCGGGCTTTCAAGTACCTG-3′	this study
bisdBdown-R	5′-AACGACGGCCAGTGCCAGGAAGGCGAGTTTCCTATAG-3′	this study

[a] Nit^r, nitrofurantoin resistant; [b] Kan^r, kanamycin resistant; [c] Tc^r, tetracycline resistant; [d] the restriction sites in the primers (5′→3′) are underlined.

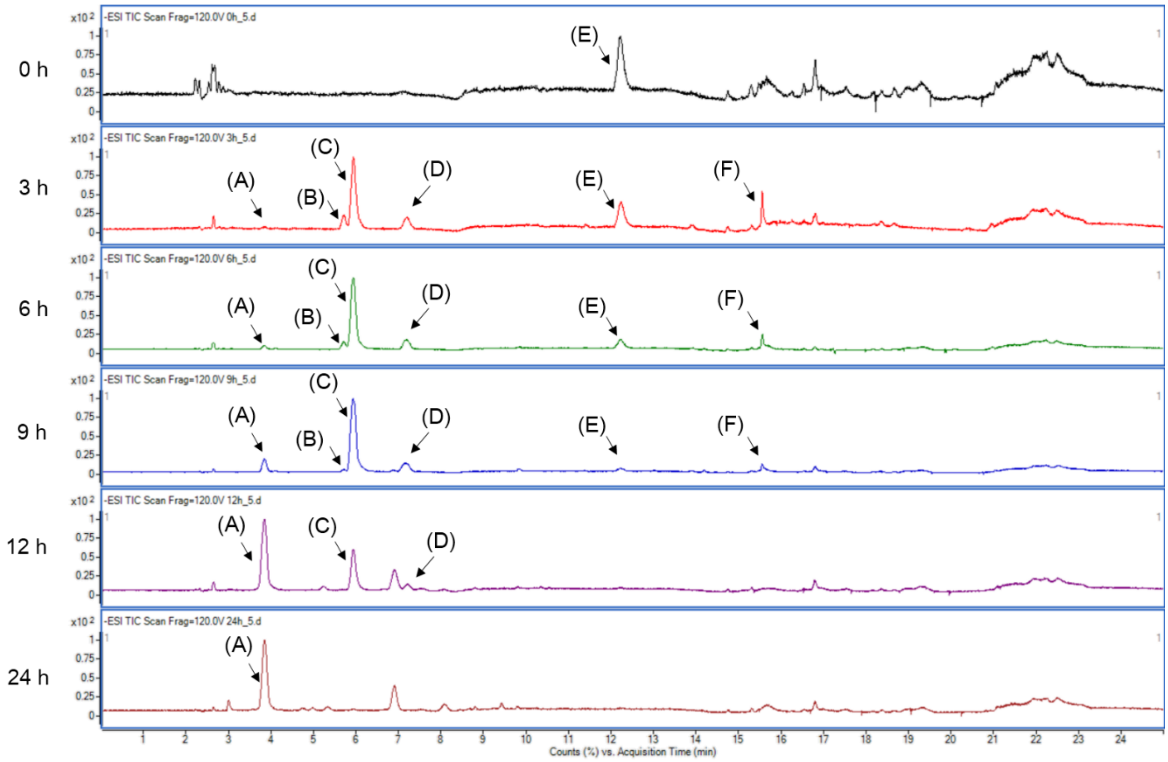

Fig. S1. HPLC-TOF-MS/MS chromatograms of intermediates during the biodegradation of BPA from 0 h to 24 h. The retention times of the identified peaks are 3.873 min (A), 5.709 min (B), 5.946 min (C), 7.203 min (D), 12.236 min (E), and 15.560 min (F).

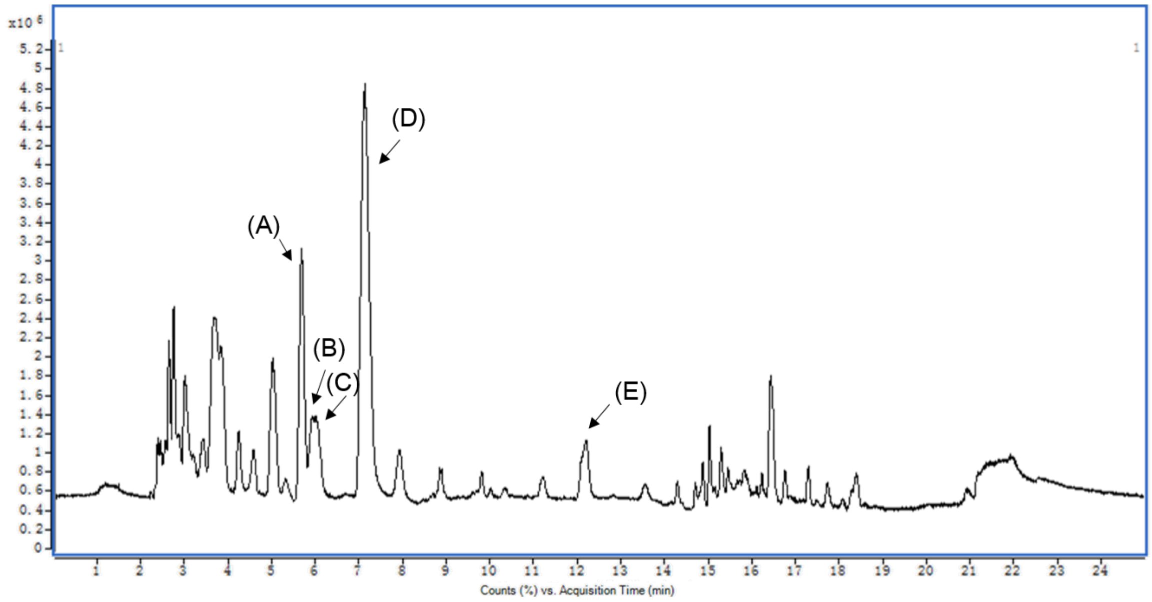

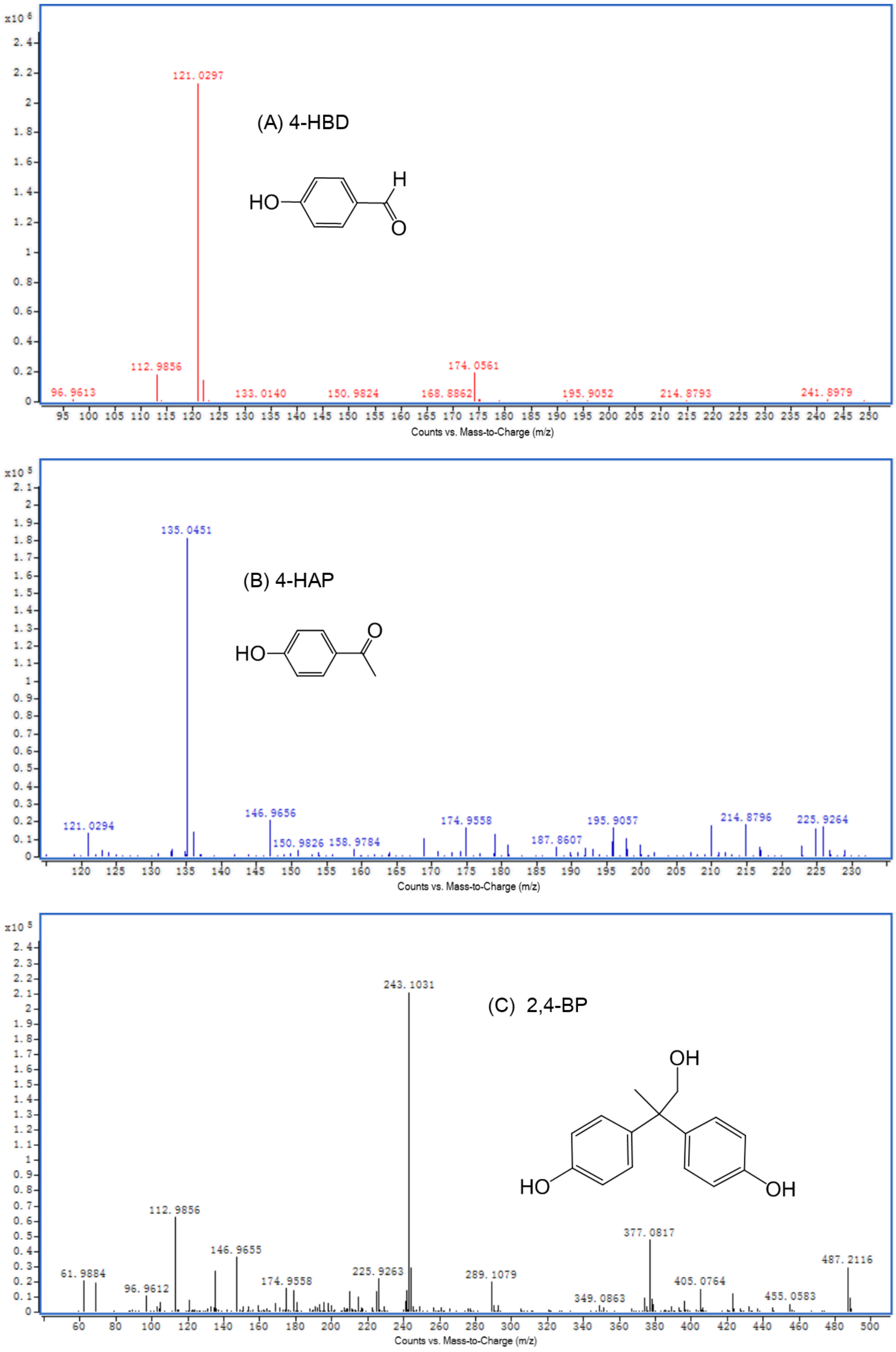

(A) 4-HBD
x10 6
121.0297
112.9856
174.0561
96.9613
133.0140
150.9824
168.8862
195.9052
214.8793
241.8979
Counts vs. Mass-to-Charge (m/z)
(B) 4-HAP
x10 5
135.0451
121.0294
146.9656
150.9826
158.9784
174.9558
187.8607
195.9057
214.8796
225.9264
Counts vs. Mass-to-Charge (m/z)
(C) 2,4-BP
x10 5
243.1031
112.9856
146.9655
61.9884
96.9612
174.9558
225.9263
289.1079
349.0863
377.0817
405.0764
455.0583
487.2116
Counts vs. Mass-to-Charge (m/z)

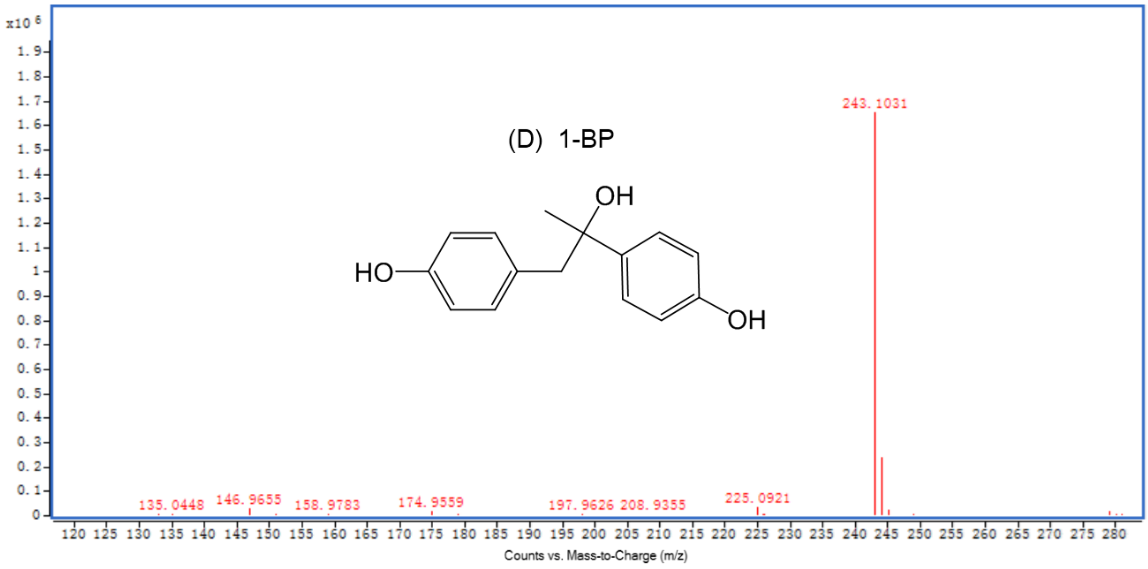

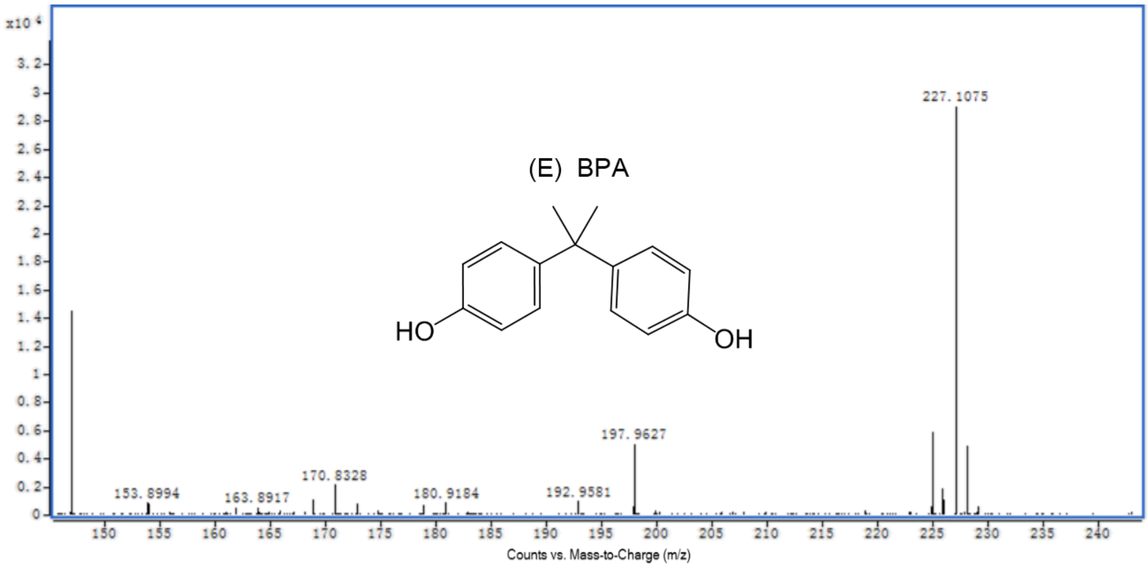

Fig. S2. The chromatogram and mass spectra of primary ions for BPA degradation by *E. coli* (pET28a-*bisdAB*) intermediates analyzed by HPLC-TOF-MS/MS.

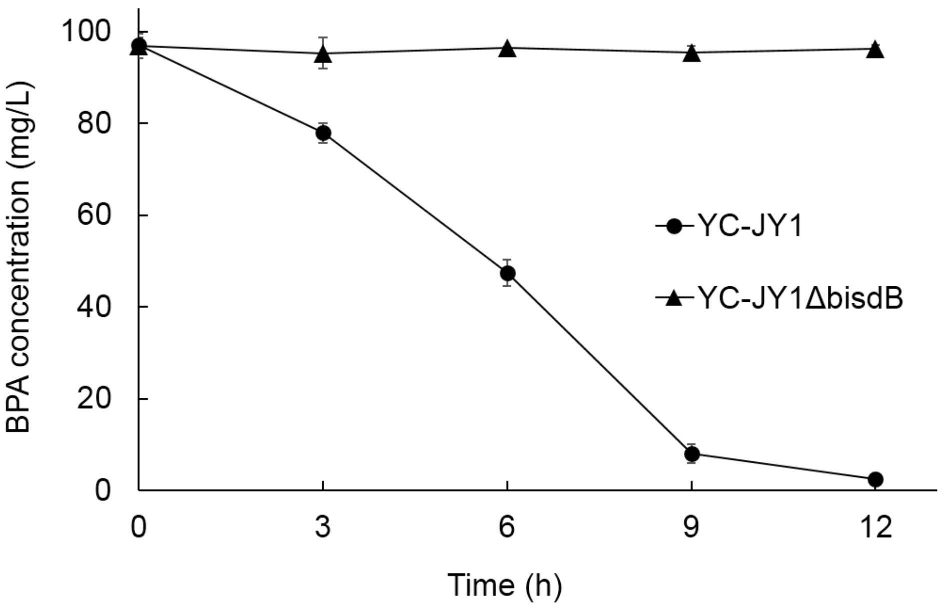

Fig. S3. BPA degradation by strain YC-JY1 and strain YC-JY1Δ*bisdB* in TEM medium.
